# Infective Endocarditis Presenting With Non-ST Elevation Myocardial Infarction: A Case Report

**DOI:** 10.7759/cureus.47147

**Published:** 2023-10-16

**Authors:** Anthony J Dina, Sherell Hicks, Charles Khoury

**Affiliations:** 1 Emergency Medicine, University of Alabama at Birmingham School of Medicine, Birmingham, USA

**Keywords:** non-st segment elevation myocardial infarction (nstemi), case report, staphylococcus hominis, acute coronary syndrome, infectious endocarditis

## Abstract

Infectious endocarditis (IE) is a rare disease characterized by infection of the endocardial surface of the heart. IE predominately involves the left-sided valves; however, right-sided valvular IE has increased in incidence with intravenous drug use. Treatment of IE is centered on targeted antibiotic therapy and management of complications, including septic embolization, which can affect all of the major arterial beds. Acute coronary syndrome secondary to septic embolization can be difficult to identify and carries an increased risk of morbidity and mortality. Care is further complicated by a lack of formal guidelines from any organization to inform management. We present a case of *Staphylococcus hominis* endocarditis complicated by coronary artery embolization and non-ST elevation myocardial infarction at the time of presentation to the emergency department, followed by a discussion of available treatment modalities.

## Introduction

Infectious endocarditis (IE) is characterized by infection of the endocardial surface of the heart and can involve native or prosthetic heart valves, intracardiac structures, and non-functional embryonic remnants in the right atrium [1]. IE is a rare disease with an incidence of 15/100,000 people in the United States [2]. It exhibits a predisposition for left-sided valvular involvement, accounting for 90%-95% of all cases. However, right-sided IE is more commonly associated with intravenous drug use (IVDU), intracardiac devices, and central venous catheters [3,4]. The most common inciting pathogen in the United States is *Staphylococcus aureus* (31%); however, other causative organisms include *Streptococcus viridans* (17%), coagulase-negative *Staphylococci* (CoNS) (11%), *Enterococci* (11%), *Streptococcus gallolyticus* (formerly *Streptococcus bovis*) (7%), other *Streptococci* (5%), *Haemophilus*, *Aggregatibacter*, *Cardiobacterium*, *Eikenella*, and *Kingella* (HACEK) organisms (2%), non-HACEK gram-negative bacteria (2%), and fungi (2%) [3]. *Staphylococcus hominis* is a coagulase-negative staphylococcus that is part of normal skin flora and is typically identified as a contaminant, but on rare occasions it can cause IE [5]. Prompt treatment of IE relies on empiric antibiotic therapy followed by identification of the causative organism via blood cultures and directed antibiotic therapy to prevent embolization. Embolization is due to dislodgement of the vegetation, resulting in hematogenous spread to other organs. Embolization occurs in 22% to 50% of cases and often involves major arterial beds, including the brain, lungs, heart, spleen, bowel, and extremities, with central nervous system embolization being most common (65% of cases). It can occur at any point before or after the initiation of therapy; however, the rate of embolization decreases with treatment [6].

We report a case of *S. hominis* aortic valve endocarditis related to IVDU in a 42-year-old man, which was complicated by septic embolization to the coronary arteries and resulted in non-ST elevation myocardial infarction (NSTEMI) at the time of presentation.

## Case presentation

A 42-year-old man with a past medical history of IV methamphetamine and heroin use presented to the emergency room for evaluation of chest pain with associated bilateral arm pain, nausea, shortness of breath, and subjective fevers, which began on the day of presentation. At the time of arrival, the patient was noted to be afebrile (99.2), tachycardic (122 bpm), and hypotensive (87/51). The physical examination was remarkable for Janeway lesions on the fingertips and right lower lobe coarse breath sounds. The patient was breathing comfortably with symmetrical peripheral pulses and no JVD, murmur, or rub. The patient exhibited no signs of peripheral edema.

Initial lab examination revealed hyponatremia, elevated creatinine, elevated BUN, elevated troponin, and low hemoglobin (Table 1).

**Table 1:** Abnormal lab values

Test	Value	Reference Range
Sodium	132 mMol/L	133-145 mMol/L
Creatinine	3.2 mg/dL	0.7-1.3 mg/dL
Bun	64 mg/dL	5-22 mg/dL
Troponin	12695 ng/L	3-20 ng/L
Hemoglobin	9.9 gm/dL	13.5-17 gm/dL

**Figure 1:**
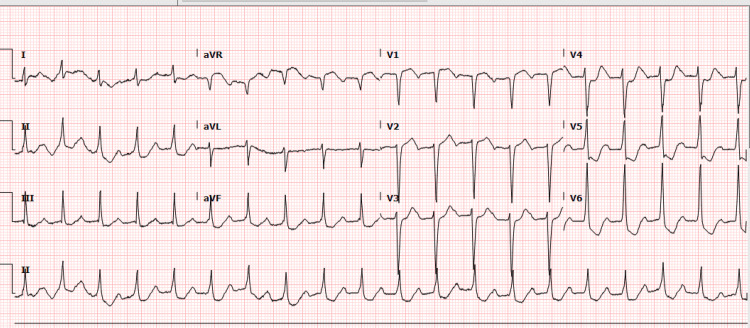
Echocardiogram at presentation with ST depression in the inferolateral leads and less than 1 mm ST elevation in V1 and aVR

The electrocardiogram (EKG) revealed sinus tachycardia at a rate of 117 bpm, prolonged PR interval, normal axis, ST depressions in the inferolateral leads, and less than 1 mm ST elevation in V1 and aVR (Figure 1).

**Figure 2:**
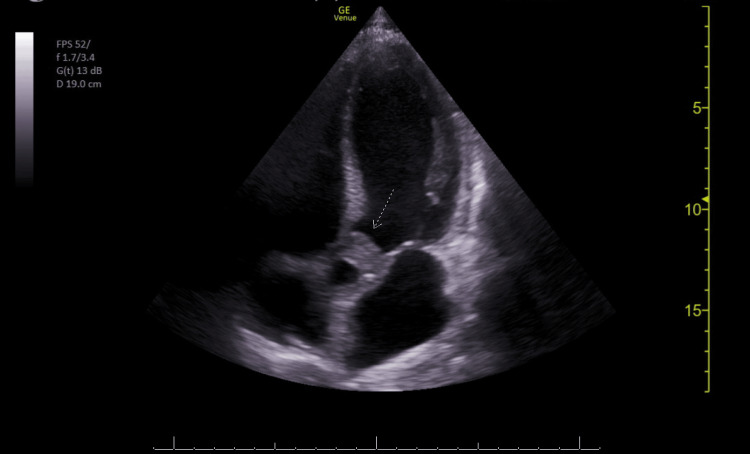
Point-of-care echocardiogram with thickened aortic valve

Three sets of blood cultures were drawn. Chest X-rays revealed mild cardiomegaly and bilateral (right greater than left) airspace opacities with a basilar predominance. A point-of-care echocardiogram revealed a reduced left ventricular ejection fraction, global left ventricular hypokinesis, and a thickened aortic valve with mild aortic insufficiency (Figure 2).​​​​​​​

**Figure 3:**
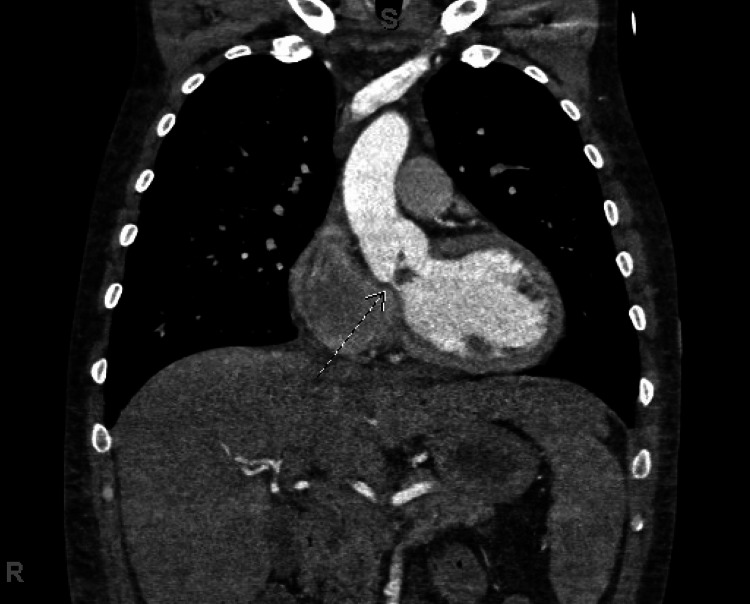
Aortic valve vegetation on computed tomography angiography of the chest

Computed tomography (CT) angiography of the chest, abdomen, and pelvis was remarkable for valvular endocarditis of the left aortic cusp, pulmonary edema, and scattered ground glass. Consolidative and nodular opacities were concerning for superimposed infection, and non-specific peripheral opacities were concerning for septic pulmonary emboli (Figure 3).

The patient remained hypotensive despite fluid resuscitation with two liters of lactated Ringer’s solution. A norepinephrine drip was started. The patient received empiric broad-spectrum coverage with vancomycin and cefepime in the emergency room. Cardiology was consulted, and the decision was made to defer PCI, start a heparin drip, and admit the patient to the cardiac care unit with plans to consult cardiovascular surgery for valve repair. 

After admission, the patient developed progressively worsening tachypnea and shortness of breath without hypoxia or hypercapnia. He subsequently suffered cardiac arrest with an intra-arrest rhythm of pulseless electrical activity approximately 12 hours into his admission. Transient return of spontaneous circulation (ROSC) was obtained twice, but the patient ultimately had recurrent cardiac arrest and expired. EKG performed during the first ROSC revealed a new left bundle branch block. Blood cultures were later positive for *S. hominis*.

## Discussion

Acute coronary syndrome (ACS) is a recognized but rare complication of IE and occurs in 2.9% of patients, with <1% presenting with ST elevation myocardial infarction [7,8]. It typically occurs within the first 15 days of presentation, with a clinical history and course similar to atherosclerotic coronary disease [7]. Because of this, a high index of suspicion must be maintained in the patient presenting with cardiac symptoms, elevated troponin, or EKG changes with a concurrent history concerning endocarditis.

In 85.7% of cases, the vegetation involves the aortic valve. These cases of ACS are due to extrinsic compression of the coronary arteries secondary to peri-annular complications, with the minority occurring due to septic embolization. When embolization does occur, it most frequently affects the left anterior descending artery [9].

Treatment of ACS in the setting of IE is controversial, with no guidelines or consensus in the literature. Potential treatments are largely based on case report data, and have included valve replacement with concurrent surgical embolectomy, percutaneous coronary intervention (PCI) angioplasty with or without stent placement, aspiration thrombectomy, and thrombolytics [9-12]. Thrombolytics carry an increased hemorrhage risk in IE, likely due to silent embolization of other arterial beds. In one study, the intracranial hemorrhage rate was as high as 80% [10,13]. PCI has better outcomes than thrombolytics, although the efficacy of treatment has not been established, and intervention also carries a risk of mycotic aneurysm formation at the site of angioplasty, displacement of the embolus distally, and infectious seeding of the stent [9,10]. Aspiration thrombectomy demonstrates good outcome data and may avoid the complications above [9]. Regardless of the selected treatment modality, infection control with broad-spectrum antibiotics followed by targeted antibiotic therapy is an integral part of the treatment.

## Conclusions

A high index of suspicion for IE-associated ACS should be maintained in patients presenting with a clinical history and lab findings concerning ACS with concurrent risk factors and a history consistent with IE. Thrombolytics should be avoided because of the increased risk of hemorrhage. Treatment should emphasize early antibiotic therapy with a multidisciplinary approach to determine the best intervention for the management of ACS. 
